# Cutaneous Mycobacterium Marinum Infection (Fish Tank Granuloma) in a Renal Transplant Recipient: Case Report and Literature Review

**DOI:** 10.7759/cureus.6013

**Published:** 2019-10-28

**Authors:** Ahmad Assiri, Sylvie Euvrard, Jean Kanitakis

**Affiliations:** 1 Dermatology, Edouard Herriot Hospital, Lyon, FRA

**Keywords:** atypical mycobacterioses, mycobacterium marinum, organ transplantation, immunosuppression, skin infection, doxycycline

## Abstract

Atypical mycobacterioses are unusual infections of the skin and other organs caused by non-tuberculous mycobacteria. Fish tank granuloma and swimming pool granuloma are two forms of atypical mycobacterioses caused by Mycobacterium marinum. So far, only a few cases of these infections have been reported in organ transplant patients, and these usually are more severe when compared with atypical mycobacterioses in immunocompetent hosts. We report a kidney transplant patient with a rather mild form of atypical mycobacteriosis (fish tank granuloma) who responded well to treatment with doxycycline and will provide a review of all similar cases reported in the literature.

## Introduction

Organ transplant recipients (OTRs) are at increased risk to develop infectious diseases because of the immunosuppressive treatment administered to prevent graft rejection. Atypical mycobacteriosis (AM) is a rare infection due to the non-tuberculous mycobacteria species. One of them, Mycobacterium marinum (M. marinum), is the cause of two clinical forms of AM, namely, fish tank (or aquarium) granuloma and swimming pool granuloma [1-2]. AM has rarely been reported in OTRs. We report here a renal transplant recipient who developed fish tank granuloma and briefly review the salient features of AM due to M. marinum in this group of patients.

## Case presentation

A 72-year-old Caucasian male received a renal allograft at the age of 62 years because of liver-kidney polycystic disease and was thereafter treated with cyclosporin (200 mg/d), steroids (5 mg/d), and mycophenolic acid (720 mg/d). His post-transplant course was complicated by cutaneous warts, multiple actinic keratoses on sun-exposed areas, a squamous cell carcinoma on each ear, and porokeratosis of the shin. Nine years post-graft, he developed a rapidly-growing cutaneous lesion over the right index finger, for which his family physician prescribed an antibiotic treatment (amoxicillin/clavulanic acid, 3 g/d, and local fusidic acid ointment). However, this treatment proved ineffective; therefore, the patient was referred to our specialized outpatient clinic devoted to the care of cutaneous complications in OTR.

**Figure 1:**
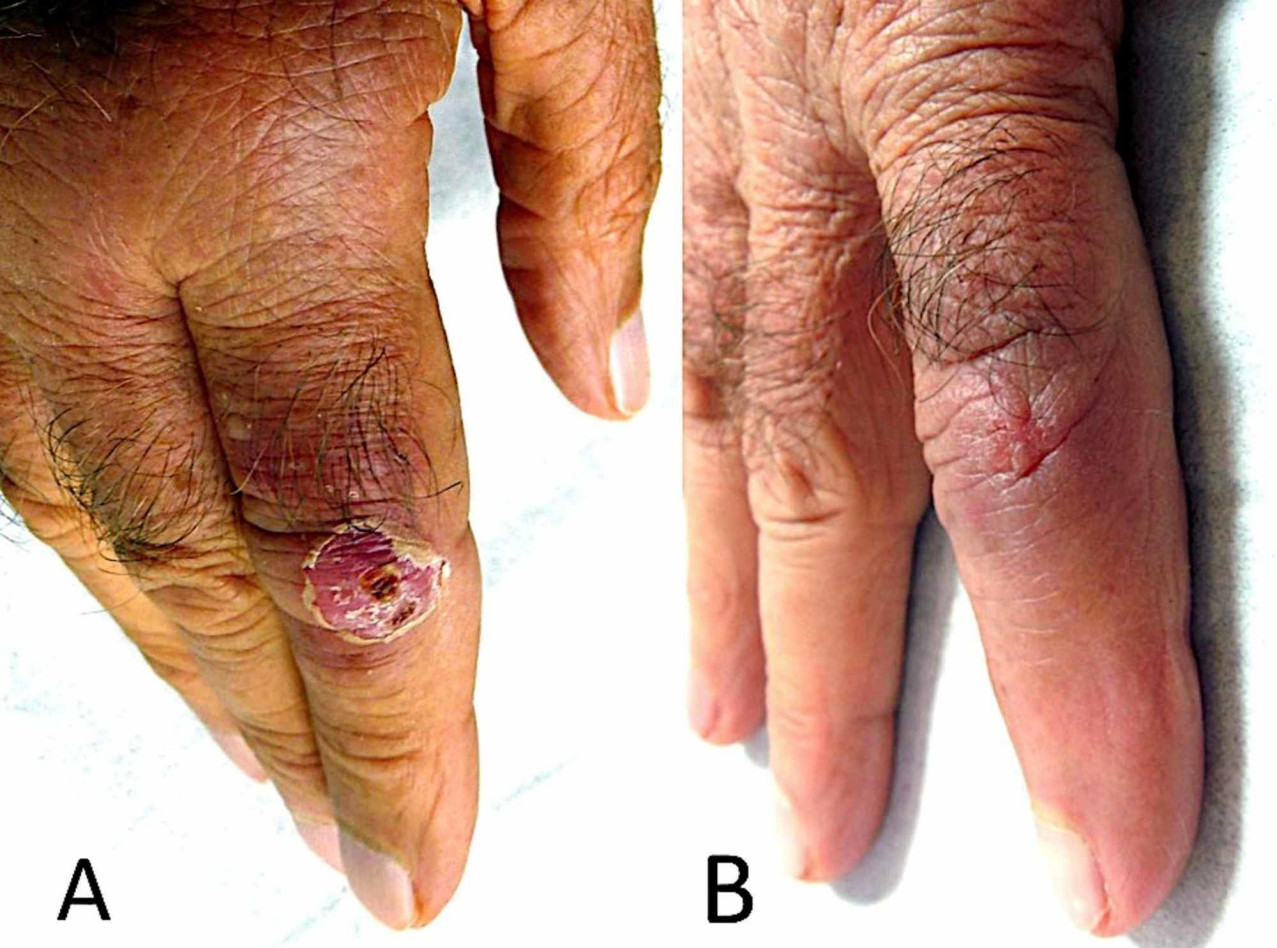
Asymptomatic erythematous, scaly nodule over the proximal interphalangeal joint of the right index finger A) An erythematous, scaly nodule over the right index finger; B) Almost complete regression of the lesion after a two-month treatment with doxycycline.

On admission, physical examination revealed an asymptomatic erythematous, scaly nodule over the proximal interphalangeal joint of the right index finger (Figure 1A). Detailed history revealed a minor trauma (cut) suffered by the patient while cleaning his fish tank one week prior to the disease onset. This fact was highly suggestive of the diagnosis of fish tank granuloma. Therefore, a biopsy was taken from the lesion under local anesthesia for histologic and bacteriologic examination.

**Figure 2:**
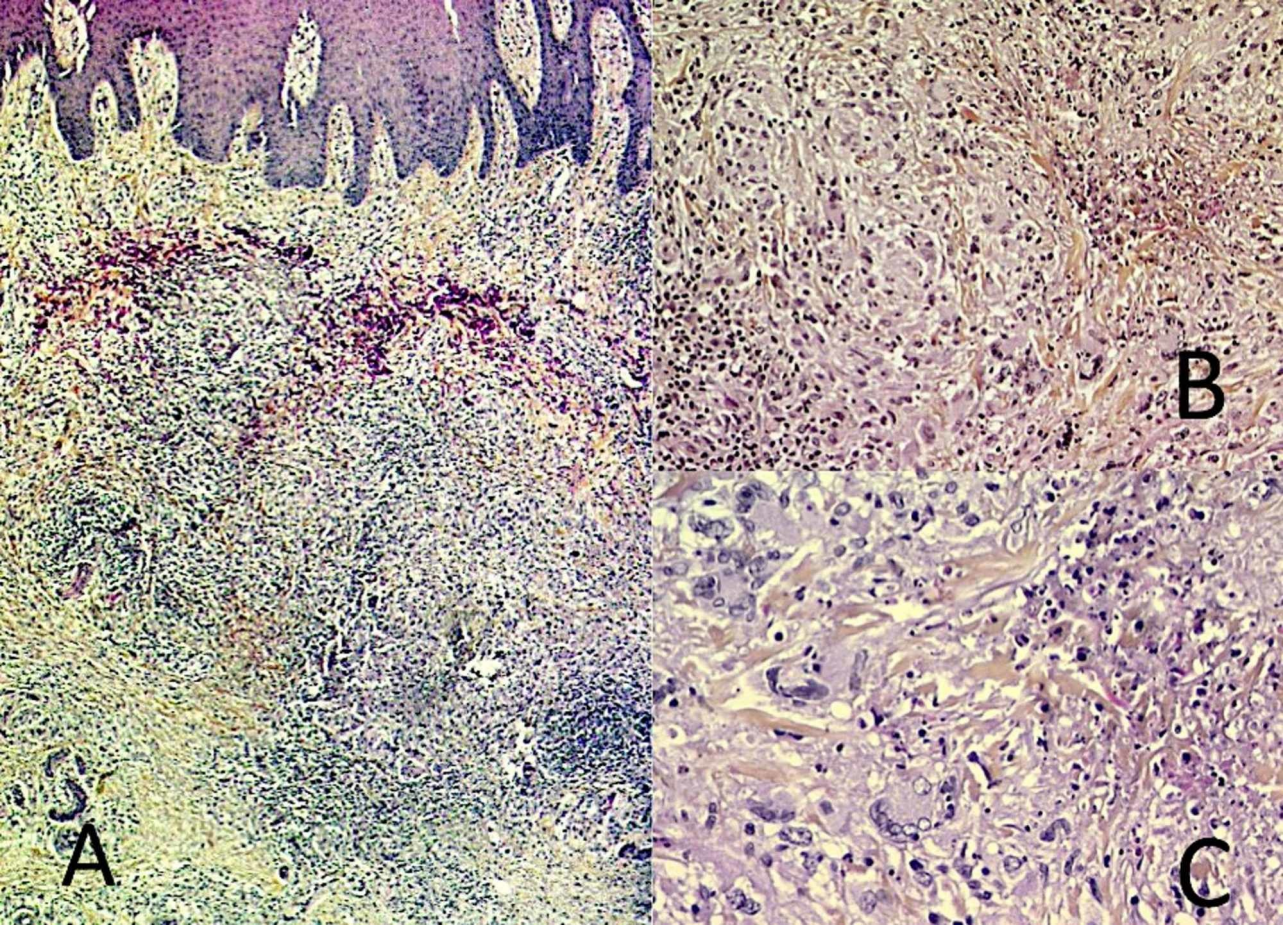
Microscopic examination of the skin biopsy A) Histological examination of the lesion shows a hyperplastic epidermis and a dense cell infiltrate in the dermis; B-C) On higher magnification, the infiltrate consists of lymphocytes, macrophages, neutrophils, and multinucleated giant cells. The dermis contains granular, basophilic, pre-necrotic areas.

Microscopic examination of the skin biopsy showed a hyperplastic epidermis and a dense polymorphous dermal infiltrate made of lymphocytes, macrophages, neutrophils, and multinucleated giant cells, occasionally surrounding pre-necrotic foci in the mid-dermis (Figure 2A-C).

Microscopic examination at high magnification of tissue sections stained with periodic acid-Schiff (PAS) and Ziehl stains did not reveal microorganisms. The culture of the biopsy specimen was negative; however, polymerase chain reaction (PCR) (post-amplification sequencing of the heat-shock protein 65 gene (hsp65)) revealed an amplicon characteristic of M. marinum (or ulcerans), thereby confirming the diagnosis of fish tank granuloma. Before the results of the bacteriological examination were available, the patient was prescribed minocycline, 200 mg/d. Almost complete regression of the lesion was noted after one month (Figure 1B). After a second month on minocycline, the patient was switched to doxycycline (200 mg/d), which he received for an additional three months. The treatment resulted in complete regression of the lesion, although the patient reported some stiffness and a decrease of skin sensitivity of his index finger. The immunosuppressive treatment of the patient was reduced (cyclosporin dosage was lowered to 125 mg/d); however, this was followed by a slight decrease in his renal function.

## Discussion

Fish tank granuloma is a clinical form of AM due to M. marinum. This microorganism was first isolated from carcasses of saltwater fishes in the aquarium of Philadelphia in 1926 [3]. Its role in tuberculosis of freshwater (platy) fishes was recognized in 1942 and the microorganism was named M. platypoecilus [4]. In 1951, the first observations of human infection (swimming pool granuloma) were reported in swimmers in contaminated pools in Sweden, and the responsible agent was named M. balnei [5]. It was later recognized that M. balnei and M. platypoecilus were identical, and they were renamed M. marinum [6].

M. marinum is a slow-growing, non-tuberculous mycobacterium belonging to the Runyon group I, requiring seven to 10 days to grow when cultured at 30° - 33°C [2, 6]. It has a worldwide distribution but is mainly found in temperate climates in stagnating water (such as swimming pools and fish tanks but also in ponds, rivers, beaches, mud, and the sea). The hosts include saltwater or freshwater fishes, snails, dolphins, and shellfishes.

M. marinum is pathogenic in traumatized, abraded skin, although a history of trauma is not always recorded by the patients. Infection occurs in contaminated swimming pools, by handling infected fish, or following (minor) trauma in an aquarium, even though this may pass unnoticed. The usual incubation period is two to six weeks but may take as long as nine months [7].

Cutaneous infections due to M. marinum have been mainly reported in immunocompetent hosts [8-9] but have been also rarely reported in OTR. Our literature review revealed 11 such cases (including the one presented here); they included five patients with renal, two with combined pancreas and kidney, two with lung, one with liver, and one with hematopoietic stem-cell transplantation [10-19] (Table 1).

In these transplantation-associated cases, there was no gender predilection (6 women/5 men). The mean age of patients was 50 years (range: 30 - 72), and the mean delay of disease onset after the (latest) transplantation procedure was 2.9 years (range: 5 months - 9 years). A history of exposure to fish or fish tank water was mentioned in nine cases.

**Table 1:** Cases of Mycobacterium marinum Skin Infections in Organ Transplant Recipients (OTR) Aza: azathioprine; Az: azithromycin; bid: twice a day; Cip: ciprofloxacin; Clar: clarithromycin; CyA: cyclosporin; Dxc: doxycycline; Eth: ethambutol, F: female; INH: isoniazid; IVIg: intravenous immunoglobulins; K: kidney; L: left; Lu: lung; Li: liver; M: male; Mi: minocycline; MMF: mycophenolate mofetil; Mox: moxifloxacin; OD: once a day; P: pancreas; Post-tx: post-treatment; Pred: prednisone; R: right; Rif: rifampicin; Tac: tacrolimus; Tmp-Smx: trimethoprim-sulfamethoxazole; tx: transplantation

Case/ Ref #	Age/ Gender	Organ Transplant /Treatment	Transmission	Disease onset post-tx (delay)	Clinical appearance	Treatment	Outcome
1 [10]	30/M	K/Aza, Pred	fish tank	3 years	Linear, tender, ulcerated nodules on extremities	Eth, 1 g/day, + Rif, 600 mg/day - recurrence after Rif was stopped, retreated with Eth +Tmp-Smx	not reported
2 [11]	52/F	K/CyA, Aza	fish tank	1.5 years	Sporotrichoid nodules on the lateral aspect of R fifth finger, hand, forearm, and arm	Doxy, 400 mg/day, but progressed, then Rif, 600 mg/day, + Eth, 800 mg/day for 3 months	cure
3 [12]	48/M	K/CyA, MMF, Pred	fishing, swimming	1 year	Linear, tender, ulcerated, 1 - 2 cm nodules on L forearm	Eth, 1.2 g/day, + Cip, 750 mg bid for 6 months, recurrence treated with 3 additional months of Emb + Cip	cure
4 [16]	52/F	Lu/CyA, Aza, Pred	fish tank	2.5 years	Tender, firm, < 1.5 cm nodules on R hand and forearm	Eth, 800 mg/day, + Az, 500 mg/day, + Minoc, 100 mg bid for 6 months: surgical excision of lesions because of tissue irritation	cure
5 [15]	45/F	P-K/CyA, Aza	unknown	8 years	Ulcerated, tender 3 cm nodules on arms and L foot	Eth, Rif, INH, addition of protionamide total treatment 4 months	cure (died of cerebral hemorrhage)
6 [14]	37/M	K-P/Tac, MMF, Pred	fish tank	1.5 years	Erythematous, tender, ulcerated nodules in ascending pattern on L forearm	Clar, 500 mg bid, + Rif, 300 mg/day	improved at 4 months of ongoing therapy
7 [13]	50/F	K/Tac, MMF, Pred	unknown	3 months after latest (3rd) tx	Sporotrichoid, ulcerated painful nodules on L arm	Triple tuberculostatic therapy for 3 months	Lesions subsided
8 [18]	50/F	Li/Tac, MMF, Pred	contact with marine fish (fishing)	2.5 years	Multiple painless ulcerated nodules on the nose spreading to face, upper limbs, and R leg	Rif, 300 mg OD, Eth, 800 mg every 3 days, and Mox, 400 mg OD, for 1 year	Cure
9 [19]	63/M	Stem cell/ cytarabine, idarubicin, busulfan, melphalan, fludarabine, anti-thymocyte globulin	fish tank	5 months	Diffuse sporotrichoid nodules on upper and lower limbs, scrotum, forehead – multiorgan involvement	Clar, Rif, tigecycline, switched to Clar, Rif, and Eth for one year	Improved at 6 months of ongoing therapy
10 [17]	52/F	Lu/Tac, Pred, MMF IVIg, rituximab for rejection 3 months post-tx	fish tank	2.5 years	Erythematous, tender nodules of distal extensor of forearm, 2 proximal subcutaneous nodules in sporotrichoid distribution	Clar, 500 mg bid, + Eth, 900 mg OD	Improved at 6 months of treatment
11 (this case)	72/M	K/CyA, Pred, MMF	fish tank	9 years	Single painless nodule on the R index finger	Dxc, 200 mg bid	Cure after one month

Clinically, cutaneous M. marinum infection manifests with nodules or pustules that may progress to ulcers, abscesses, or warty plaques [1, 8-9]. It may spread proximally along the lymphatic vessels in a sporotrichoid pattern, mimicking sporotrichosis [13-14]. The lesions predominate on the extremities since the growth of the microorganism is inhibited at 37°C. Swimming pool granuloma develops on trauma-prone body zones, such as the elbows, knees, feet, and tip of the nose, whereas fish tank granuloma predominates on the dominant hand (as in our patient). Locoregional lymphadenopathy is usually absent. The infection may extend to deeper tissues, causing tenosynovitis, osteomyelitis, septic arthritis, and may even disseminate to internal organs, especially in immunocompromised patients [1, 20].

OTR with AM presented with multiple, occasionally painful, nodular or pustular lesions with a tendency to ulcerate and to extend locally in an ascending sporotrichoid pattern (Table 1). The lesions affected the upper extremities (75% of cases) and less often in the lower limbs (four cases, i.e., 36%), possibly as a result of disease dissemination [10, 15, 18-19].

A documented systemic dissemination was reported in a patient with hematopoietic stem cell transplantation for acute myeloid leukemia followed by two courses of aggressive chemotherapy [19]. Apart from cutaneous lesions (which predominated on the lower limbs), the patient also had intramuscular and intraosseous involvement (shown on MRI) and hypermetabolic lesions seen on positron emission tomography (PET) scan in the brain, lung, scalp, face, nasal cavity, scrotum, and testis. Despite the widespread disease, the lesions improved after six months of treatment, given for one year. 

Three patients had multiple skin lesions over several body sites, possibly as a result of systemic dissemination (rather than to multiple sites of inoculation) [10, 15, 18]. Our patient differed from these cases as he had a mild disease (limited to a single asymptomatic nodule) which developed rather late (nine years) after transplantation.

The diagnosis of AM can be suspected by history (contact with fishes or with a fish tank, swimming in contaminated swimming pools) and is confirmed by isolation of the responsible microorganism from the lesions. This can be achieved with cultures, which are positive in 70% - 80% of cases, and also allows for an antibiogram to be performed [1]. Histologically, the lesions are characterized by a non-caseating granulomatous skin infiltrate. Mycobacterial microorganisms are seldom found, even after specific (Ziehl) histochemical stains, as in our case. Of note, a monoclonal antibody to M. marinum, detecting an antigen of 56-kDa, is available for immunohistochemical use on tissue sections. PCR with specific primers appears as the most sensitive and specific diagnostic method [9] and allowed diagnostic confirmation in the case of our patient.

The treatment of cutaneous infections due to M. marinum is not standardized and depends on several factors, including mainly the severity of the infection and the immune status of the patient. In immunocompetent patients, the lesions are often superficial and may regress spontaneously or after antibiotic monotherapy [2]. In immunocompromised hosts, such as OTR, the infection is usually more severe; therefore, treatment with at least two antibiotics is advised so as to avoid the development of resistance. Clarithromycin and trimethoprim-sulfamethoxazole are both efficacious and safe. Cyclins (minocycline, doxycycline) are also effective at a dose of 200 mg/d. Cyclins may be combined with rifampicin or ethambutol in severe cases or in the setting of immunosuppression [2, 8-9]. Clarithromycin and rifampicin, clarithromycin and ethambutol, or ethambutol and rifampicin in combination have also been used with good results in such cases [20]. On average, the duration of the treatment is three months or six weeks after clinical healing [2], but a much longer treatment duration (up to 12 months) may be necessary in case of a deep extension or dissemination [2, 18-19]. In OTR, M. marinum infections were treated with at least two antibiotics with rare exceptions (such as our patient, who had mild disease). In another OTR treated with doxycycline as monotherapy, progression was noted, prompting a switch to rifampicin and ethambutol [13]. Ethambutol was the most common antibiotic used in OTR (nine cases), followed by rifampicin (seven cases), and clarithromycin (three cases). The most commonly used drug combination was ethambutol and rifampicin (five patients), including all four cases of disseminated infection. Other antimicrobials used included azithromycin, ciprofloxacin, moxifloxacin, isoniazid, protionamide, and trimethoprim-sulfamethoxazole (Table 1). In addition to antimicrobial treatment, deep lesions may be surgically excised. Prevention of an AM infection includes the use of gloves when cleaning fish tanks, rapid disinfection of wounds, and adequate chlorination of swimming pools. 

## Conclusions

Atypical mycobacterioses of the skin are unusual infections, even in organ transplant recipients in whom they usually manifest with more severe signs compared with immunocompetent patients. In that respect, the case of our renal transplant patient with fish tank granuloma due to Mycobacterium marinum was unusual as the disease was mild and could be treated effectively with doxycycline as monotherapy. The diagnosis of AM is not straightforward, but a detailed history (namely, concerning occupation, hobbies, and activities) helps to identify such cases.

## References

[1] Bonamonte D, De Vito D, Vestita M, Delvecchio S, Ranieri LD, Santantonio M, Angelini G (2013). Aquarium-borne Mycobacterium marinum skin infection. Report of 15 cases and review of the literature. Eur J Dermatol.

[2] Rallis E, Koumantaki-Mathioudaki E (2007). Treatment of Mycobacterium marinum cutaneous infections. Expert Opin Pharmacother.

[3] Aronson D (1926). Spontaneous tuberculosis in salt water fish. J Infect Dis.

[4] Baker A, Hagan WA (1942). Tuberculosis of the Mexican platyfish (Platypoecilus maculatus). J Infect Dis.

[5] Greenberg AE,  Kupka E (1957). Swimming pool injuries, mycobacteria, and tuberculosis-like disease. Public Health Rep.

[6] Gluckman SJ (1995). Mycobacterium marinum. Clin Dermatol.

[7] Jernigan J, Farr B (2000). Incubation period and sources of exposure for cutaneous Mycobacterium marinum infection: case report and review of the literature. Clin Infect Dis.

[8] Holden IK, Kehrer M, Andersen AB, Wejse C, Svensson E, Johansen IS (2018). Mycobacterium marinum infections in Denmark from 2004 to 2017: a retrospective study of incidence, patient characteristics, treatment regimens and outcome. Sci Rep.

[9] Dodiuk-Gad R, Dyachenko P, Ziv M (2007). Nontuberculous mycobacterial infections of the skin: a retrospective study of 25 cases. J Am Acad Dermatol.

[10] Gombert ME, Goldstein EJ, Corrado ML, Stein AJ, Butt KM (1981). Disseminated Mycobacterium marinum infection after renal transplantation. Ann Intern Med.

[11] Dompmartin A, Lorier E, de Raucourt S, Vergnaud M, Ryckelynck JP, Hurault de Ligny B, Leroy D (1991). Sporotrichoid form of M. marinum infection in a patient treated with cyclosporin following kidney transplantation (Article in French). Ann Dermatol Venereol.

[12] Farooqui MA, Berenson C, Lohr JW (1999). Mycobacterium marinum infection in a renal transplant recipient. Transplantation.

[13] Lovric S, Becker JU, Kayser D, Wagner A, Haubitz M, Kielstein JT (2009). Fish, flesh and a good red herring: a case of ascending upper limb infection in a renal transplant patient. Clin Nephrol.

[14] Pandian TK, Deziel PJ, Otley C, Eid AJ, Razonable RR (2008). Mycobacterium marinum infections in transplant recipients: case report and review of the literature. Transpl Infect Dis.

[15] Schmekal B, Janko O, Zazgornik J, Schinko H, Bogner S, Syre G, Biesenbach G (2002). Skin tuberculosis with atypical mycobacteria 8 years after combined pancreas and kidney transplantation. Am J Nephrol.

[16] Torres F, Hodges T, Zamora MR (2001). Mycobacterium marinum infection in a lung transplant recipient. J Heart Lung Transplant.

[17] Javvaji SR, Javvaji S, Grossman ME (2015). Sporotrichoid Mycobacterium marinum infection after lung transplantation for alpha-1 antitrypsin deficiency. J Surg Transplant Sci.

[18] Lau SK, Gurrem SO, Ngan AH, Yeung CK, Yuen KY, Woo PC (2011). First report of disseminated mycobacterium skin infections in two liver transplant recipients and rapid diagnosis by hsp65 gene sequencing. J Clin Microbiol.

[19] Jacobs S, George A, Papanicolaou GA, Lacouture ME, Tan BH, Jakubowski AA, Kaltsas A (2012). Disseminated Mycobacterium marinum infection in hematopoietic stem cell transplant recipient. Transplant Infect Dis.

[20] Nguyen HH, Fadul N, Ashraf MS, Siraj DS (2015). Osteomyelitis Infection of Mycobacterium marinum: A Case Report and Literature Review. Case Rep Infect Dis.

